# A Novel Gene Amplification Causes Upregulation of the PatAB ABC Transporter and Fluoroquinolone Resistance in Streptococcus pneumoniae

**DOI:** 10.1128/AAC.04858-14

**Published:** 2015-05-14

**Authors:** Alison J. Baylay, Alasdair Ivens, Laura J. V. Piddock

**Affiliations:** a Antimicrobials Research Group, School of Immunity and Infection, Institute of Microbiology and Infection, and College of Medical and Dental Sciences, University of Birmingham, Birmingham, United Kingdom; b Centre for Immunity, Infection and Evolution, Ashworth Laboratories, University of Edinburgh, Edinburgh, United Kingdom

## Abstract

Overexpression of the ABC transporter genes *patA* and *patB* confers efflux-mediated fluoroquinolone resistance in Streptococcus pneumoniae and is also linked to pneumococcal stress responses. Although upregulation of *patAB* has been observed in many laboratory mutants and clinical isolates, the regulatory mechanisms controlling expression of these genes are unknown. In this study, we aimed to identify the cause of high-level constitutive overexpression of *patAB* in M184, a multidrug-resistant mutant of S. pneumoniae R6. Using a whole-genome transformation and sequencing approach, we identified a novel duplication of a 9.2-kb region of the M184 genome which included the *patAB* genes. This duplication did not affect growth and was semistable with a low segregation rate. The expression levels of *patAB* in M184 were much higher than those that could be fully explained by doubling of the gene dosage alone, and inactivation of the first copy of *patA* had no effect on multidrug resistance. Using a green fluorescent protein reporter system, increased *patAB* expression was ascribed to transcriptional read-through from a tRNA gene upstream of the second copy of *patAB*. This is the first report of a large genomic duplication causing antibiotic resistance in S. pneumoniae and also of a genomic duplication causing antibiotic resistance by a promoter switching mechanism.

## INTRODUCTION

Gene amplification, the transient generation of tandem repeats of large chromosomal regions, is an important source of variability in bacterial populations. Data from Salmonella suggest that, in nonselected cultures, transient duplications occur at frequencies of 10^−4^ to 10^−2^, meaning that 10% to 30% of cells in a nonselected culture have a duplication somewhere in their genome at any one time (1). Due to this high prevalence, it is thought that gene amplification, rather than point mutations, provides a pool of preexisting variation allowing bacterial populations to adapt to various stresses, such as antibiotic resistance or growth on unusual carbon sources (2). However, gene amplification events are difficult to detect and study, as the tandem repeat units are identical in sequence and the gene amplifications are often unstable and are lost by recombination between repeat units once the selective pressure has been removed.

Streptococcus pneumoniae is the main bacterial cause of community-acquired pneumonia and represents a major disease burden worldwide (3). Despite the recent introduction of the heptavalent pneumococcal conjugate vaccine, antibiotic resistance is an increasing problem in this organism due to the spread of multidrug-resistant clones and increases in antimicrobial resistance among nonvaccine serotypes (4).

The *patA* (spr1887) and *patB* (spr1885) genes, which encode ABC half transporters, have been associated with the intrinsic resistance of S. pneumoniae to some fluoroquinolone antibiotics. Each half transporter consists of a nucleotide binding domain and a membrane-spanning domain, and heterodimerization of PatA and PatB is required to form a functional transporter (5). Constitutive overexpression of *patA* and *patB* has been observed in both laboratory mutants and clinical isolates and causes decreased susceptibility to ciprofloxacin and norfloxacin, as well as other agents, such as dyes and the biocide cetrimide (6–9). In all cases, *patA* and *patB* are coregulated, despite the presence of a degenerate transposase gene (spr1886), transcribed in the opposite direction, separating the two genes. Inactivation of *patA* or *patB* either genetically or using efflux inhibitors results in a hyper-antibiotic-susceptible phenotype in laboratory mutants (7) and in clinical isolates increases ciprofloxacin susceptibility even if the isolate also contains topoisomerase mutations (9).

In addition to transporting fluoroquinolones, recent evidence suggests that PatAB plays a role in pneumococcal stress responses. Fluoroquinolone transport is unlikely to be the primary physiological role of PatAB, as fluoroquinolones are synthetic antibiotics that have been in use for only 30 years. Expression of *patA* and *patB* can be induced by several fluoroquinolones, including those that are thought not to be substrates for transport, as well as the nonquinolone DNA-damaging agent mitomycin C (8, 10). This indicates that PatAB is upregulated as part of a response to DNA damage. Additionally, overexpression of *patA* and *patB* has been shown to alleviate a fitness cost of linezolid resistance (11), and a role for PatAB in response to pH stress has been suggested (12).

Despite the potential role of PatA and PatB in pneumococcal stress responses, no regulatory pathways controlling expression of *patA* and *patB* have been identified. Mutations affecting a Rho-independent transcriptional terminator upstream of *patA* were shown to cause *patAB* overexpression in three previous studies (11, 13, 14). However, mutations affecting this terminator are not found in all *patAB*-overexpressing isolates (7, 9), and in these cases the genetic changes leading to overexpression are unknown.

M184 is a mutant of the unencapsulated laboratory strain R6 (GenBank accession number AE007317.1), characterized previously by Garvey and Piddock (7). It was generated by exposure of R6 to the efflux inhibitor reserpine, followed by transformation of the reserpine-resistant phenotype of one of the selected mutants back into R6. As well as having reduced susceptibility to reserpine, M184 was found to be resistant to multiple drugs, including fluoroquinolone antibiotics, such as ciprofloxacin and norfloxacin; the dyes ethidium bromide and acriflavine; and the biocide cetrimide. This multiple-drug-resistance phenotype was shown to be due to the increased expression of *patA* and *patB*. In this study, the whole-genome sequences of M184 and three second-generation transformants were analyzed to identify mutations that might be responsible for *patAB* overexpression. This revealed a novel gene amplification which caused high levels of *patAB* expression due to a change in the genomic context of the promoter of the second copy of *patA*.

## MATERIALS AND METHODS

### Bacterial strains and growth.

All S. pneumoniae used in this study are listed in Table 1. All S. pneumoniae strains and isolates were grown statically at 37°C in an atmosphere of 5% CO_2_ in brain heart infusion (BHI) broth (Oxoid, Basingstoke, United Kingdom) or on Columbia agar (Oxoid, Basingstoke, United Kingdom) supplemented with 5% defibrinated horse blood (TCS Biosciences, Buckingham, United Kingdom). The growth of the strains in broth was assessed by measurement of the optical density at 660 nm (OD_660_) of liquid cultures and by viable cell counts.

**TABLE 1:** Bacterial strains used in this study

Strain	Description	Reference or source
R6	Unencapsulated wild-type strain derived from D39	49
M169	Spontaneous reserpine-resistant mutant	7
M184	Reserpine-resistant R6 transformant of M169	7
R6^M184^ T1	EtBr^*a*^-resistant R6 transformant of M184	This study
R6^M184^ T1	EtBr-resistant R6 transformant of M184	This study
R6^M184^ T1	EtBr-resistant R6 transformant of M184	This study
M240	R6 *patB*::*magellan2*	7
M246	R6 *patA*::*magellan2*	7

a EtBr, ethidium bromide.

Escherichia coli strains used for plasmid construction were grown overnight on Luria-Bertani (LB) agar at 37°C or in LB broth at 37°C with shaking at 180 rpm.

### DNA extraction, whole-genome sequencing, and data analysis.

Genomic DNA was extracted from all strains using a Wizard Genomic DNA extraction kit (Promega) following the manufacturer's instructions.

Genome sequencing was carried out by Genepool (Edinburgh, United Kingdom) on an Illumina HiSeq sequencing system (Illumina, Saffron Walden, United Kingdom) according to standard protocols to give 100-bp paired-end reads. Reads were mapped against the published R6 genome sequence (GenBank accession number AE007317.1) using the Bowtie2 tool (15) and the very sensitive local setting, and a consensus pileup was generated using the mpileup program from the SAMtools (version 0.1.18) suite of programs (16) and the faidx-indexed R6 genome as a reference. Raw sets of putative single nucleotide polymorphisms (SNPs) and small insertion or deletion events (indels) were generated from pileup using BCFtools (version 0.1.17-dev) and filtered to remove variants that had a quality score of less than 50 and that covered a read depth of less than 50. Identified mutations were confirmed by PCR and Sanger sequencing. To generate local assemblies, reads meeting the desired criteria were extracted from the read alignment using SAMtools and assembled with the Velvet (version 1.1.07) algorithm (17), using the VelvetOptimiser script by Simon Gladman to determine the optimal parameter settings. Read alignments were visualized, and the numbers of reads per kilobase per million reads mapped (RPKM) were calculated using the Artemis (version 14.0.0) sequence viewer (18).

### PCR and DNA sequencing.

The primer sequences and PCR conditions used in this study are listed in Table 2. PCRs were carried out essentially as described previously (7), although the Extensor PCR master mix was used to amplify the 9.2-kb duplicated region from strain M184 and its transformants.

**TABLE 2:** Details of PCR primers used in this study

Primer pair	Forward primer sequence	Reverse primer sequence	Amplicon
1	CTTATTGGTGGGGAGAAGAA	GATAACGCGGTTGCAGAAGT	Duplication junction
2	GATAGGGCAGAAGAGCATCC	GATAACGCGGTTGCAGAAGT	Intergenic region between *hexA* and *patA*
3	TCTTGCTCAGTCCATCATCGAATAT	CAGCATCGGTTCCTTGTC	*patA* copy 1
4	CTTATTGGTGGGGAGAAGAA	CAGCATCGGTTCCTTGTC	*patA* copy 2
5	ATGTTGTCCTCGCAGCCTAT	CAGCATCGGTTCCTTGTC	*patA* internal region containing *magellan2* insertion
6	TAGTATCGACGGAGCCGATT	GCTAGTTGAACGCTTCCATC	Cloning site of plasmid pBAV1K2
7	TAAGAATTCTTTAATGAGCGATAGAAGAGTCAG	GATAACGCGGTTGCAGAAGT	Duplication junction, adding a 5′ EcoRI site (underlined sequence)

PCR amplimers were sequenced by the Functional Genomics Laboratory (School of Biosciences, University of Birmingham, Birmingham, United Kingdom) as described previously (9).

### Transformation.

Transformations were carried out as previously described (7). Briefly, mid-logarithmic-phase cultures of the recipient strain were diluted 1:20 in competence medium (Todd-Hewitt broth; Oxoid, Basingstoke, United Kingdom) containing 1 mM calcium chloride (Sigma Aldrich Ltd., Dorset, United Kingdom), 0.2% bovine serum albumin (BSA; Sigma Aldrich Ltd., Dorset, United Kingdom), and 100 ng/ml competence-stimulating peptide 1 (CSP1; Mimotopes, Clayton, Victoria, Australia). Donor DNA was added at various concentrations to 500-µl aliquots of a competent cell suspension. Transformation reaction mixtures were incubated for 3 h at 37°C, and then 20-μl and 200-μl volumes were spread onto selective agar plates. Viable counts were determined in parallel to allow estimation of the transformation frequency.

### Antibiotics and susceptibility determination.

The MICs of various agents for the strains used in this study were measured using the standardized agar doubling dilution method according to the British Society for Antimicrobial Chemotherapy (19).

### Measurement of intracellular accumulation of ethidium bromide.

The efflux activities of the strains were measured by monitoring the uptake of ethidium bromide, which fluoresces when intercalated into DNA. Logarithmic-phase cultures were pelleted by centrifugation at 2,200 × *g* and resuspended in phosphate-buffered saline (PBS). Cell suspensions were adjusted to an OD_660_ of 0.1 with fresh PBS, and 100 μl cell suspension was added in triplicate to a black microtiter tray. Fluorescence was measured at excitation and emission wavelengths of 530 and 600 nm, respectively, using a FLUOstar Optima plate reader (BMG Labtech, Aylesbury, United Kingdom) every 2 min for a total of 30 min. A final concentration of 100 μM ethidium bromide was added to each well by injection on the second cycle of measurement.

### Measurement of expression of *patA* and *patB*.

RNA was extracted from three biological replicates of each strain to be tested using a Promega SV total RNA isolation kit according to the manufacturer's instructions (Promega, Southampton, United Kingdom). RNA and contaminating DNA concentrations were determined using a Qubit fluorimeter (Life Technologies, Paisley, United Kingdom). Residual DNA contamination was removed by treatment with Turbo DNase (Life Technologies), and cDNA was generated using SuperScript III reverse transcriptase (Life Technologies) following the first-strand synthesis protocol supplied by the manufacturer. Expression of *patA* and *patB* relative to the expression of *rpoB* was measured by quantitative real-time PCR by monitoring the fluorescence of SYBR green dye. Reaction mixtures consisted of 12.5 μl IQ SYBR green supermix (Bio-Rad, Hemel Hempstead, United Kingdom), 375 nM (each) forward and reverse primers, and 1 μl cDNA in a 25-μl reaction mixture. Real-time PCR was carried out using a Bio-Rad CFX96 thermal cycler with the following protocol: 3 min at 95°C, followed by 40 cycles of 10 s at 95°C and 30 s at 54.5°C. Expression values were calculated from the fluorescence data using the Pfaffl method (20).

### Insertional inactivation of individual copies of *patA* in M184.

An internal region of *patA* containing the *magellan2* minitransposon from strain M246 (R6 *patA*::*magellan2*) was amplified by PCR using primer pair 5 (Table 2). The PCR amplimer was diluted 1:10 six times, and 10 μl of each concentration was transformed into M184 as described above. Transformants were selected on 100 mg/liter spectinomycin to select for successful incorporation of the minitransposon which contains a spectinomycin resistance gene, *aad9*. Primer pairs specific for the N-terminal half of each copy of *patA* were used to determine which copy of *patA* contained the insert.

### Growth kinetics and determination of stability of gene amplification.

To measure growth kinetics, a 1% inoculum of the strain of interest was added to 50 ml BHI broth. Growth was monitored at hourly intervals by measurement of the OD_660_ for 8 h.

To measure the stability of the gene amplification, M184 cultures were grown overnight in triplicate on 8 mg/liter ethidium bromide to select for cells containing the duplication. Cells were scraped directly off the plates, resuspended in BHI broth containing 10% glycerol, and stored at −80°C. These time zero cultures were then diluted 1:1,000 in fresh BHI broth and grown exponentially without selection for 12 h (corresponding to approximately 15 generations). Samples of culture from 0 and 12 h were serially diluted in PBS and grown on nonselective agar to obtain single colonies. For each biological replicate and time point, 20 colonies were picked at random and restreaked onto both nonselective agar and plates containing 8 mg/liter norfloxacin. The proportions of colonies retaining norfloxacin resistance at 0 and 12 h were compared to calculate the probability of loss of the duplication using the following equation. Assuming that at each generation a proportion (*p*) of the population loses the duplication, the proportion of cells retaining the duplication after *n* generations is (1 − *p*)^*n*^ (21). To confirm that the loss of norfloxacin resistance correlated with loss of the duplication, DNA was extracted from a representative sample of candidate colonies and checked for the presence of the duplication junction by PCR.

### Construction of pBAV1K2 reporter plasmid, cloning of *patA* promoter and tRNA gene, and reporter assay.

To generate plasmid pBAV1K2, plasmids pBAV1K-T5-*gfp* (22) and pMW82 (23) were digested with EcoRI and PstI (Thermo Scientific), and digestion products were separated on a 1% agarose gel. The 2,800-bp plasmid backbone of pBAVK1K-T5-*gfp* and the 824-bp band corresponding to the *gfp* gene of pMW82 were extracted from the gel using a Qiagen gel extraction kit according to the manufacturer's instructions. The DNA fragments were ligated using QuickStick ligase (Bioline) and used to transform E. coli TOP10 cells (Life Technologies). Transformants were selected on 50 mg/liter kanamycin. To remove DNA methylation for subsequent digestions, the extracted plasmid was transformed into E. coli JM110 chemically competent cells (kindly provided by Yanina Sevastsyanovich) and reextracted.

To clone the *patA* promoter into pBAV1K2, giving pBAV1K2*p*, a 144-bp DNA fragment that covered the region from 12 to 146 bp upstream of the *patA* start codon and incorporated a 5′ EcoRI site and a 3′ XbaI site was synthesized by GeneArt (Life Technologies). The promoter fragment was digested with EcoRI and XbaI, purified using a QIAquick PCR purification kit (Qiagen), and combined in a 100:1 ratio with pBAV1K2 DNA linearized with XbaI and EcoRI. The DNA fragments were ligated with QuickStick ligase and used to transform E. coli TOP10 cells. Transformants were selected on 50 mg/liter kanamycin and screened for successful incorporation of the insert by PCR with primer pair 6 (Table 2). To clone the tRNA-Glu gene upstream of the *patA* promoter, giving plasmid pBAV1K2*tp*, a naturally occurring SpeI site 134 bp upstream of the *patA* start codon was exploited. A 681-bp region spanning the duplication junction in M184 was amplified using primer pair 7 (Table 2), which introduces a 5′ EcoRI site. This was digested with EcoRI and SpeI and combined in a 100:1 molar ratio with EcoRI- and SpeI-linearized pBAV1K2*p*. Ligation, transformation, transformant selection, and PCR screening were performed as described above.

Plasmid DNA was extracted from E. coli cells using a QIAquick miniprep kit according to the manufacturer's instructions, and 20 μl of each plasmid preparation was used to transform S. pneumoniae R6 as described above. Successful transformants were selected on plates containing 100 mg/liter kanamycin.

R6 cells containing pBAV1K2, pBAV1K2*p*, or pBAV1K2*tp* were grown in triplicate in BHI broth supplemented with 100 mg/liter kanamycin to an OD_660_ of 0.5. One milliliter of each culture was transferred to a microcentrifuge tube, and cells were harvested by centrifugation (14,000 × *g*, 2 min). Cells were resuspended in 200 μl sterile PBS and transferred to a black microtiter tray. Fluorescence was measured using a FLUOstar Optima plate reader (BMG Labtech) with excitation and emission wavelengths of 492 and 520 nm, respectively.

## RESULTS

### Overexpression of *patAB* can be transferred from M184 to R6 in a single transformation step.

To identify the mutation responsible for increased expression of *patA* and *patB* in M184, the genome sequences of M184 and our laboratory stock of R6 were determined by Illumina sequencing. Reads were aligned against the published R6 genome sequence to identify single nucleotide polymorphisms (SNPs) and small insertion or deletion events (indels). This analysis identified 27 point mutations and small indels present in M184 that were not found in R6 (see the supplemental material at http://epapers.bham.ac.uk/1958/). Exclusion of reads that mapped to more than one position in the genome did not change the set of variants identified (data not shown).

To separate mutations causing *patAB* overexpression from compensatory mutations and bystander mutations, M184 DNA was transformed into R6 and transformants were selected on 8 mg/liter ethidium bromide. Reserpine was not used to select transformants, as the mechanism of resistance to this agent appears to be more complex; inactivation of *patA* increased susceptibility to reserpine, but inactivation of *patB* did not (7). To avoid selecting fluoroquinolone-resistant topoisomerase mutants, ethidium bromide, another substrate of the PatAB transporter, was used to select transformants that had incorporated mutations causing *patAB* overexpression. Ethidium bromide-resistant transformants were selected in a single step, but at a transformation frequency of 10^−6^ to 10^−7^, 10- to 20-fold lower than that for transformants transformed with a spectinomycin resistance cassette under the same conditions. No spontaneous ethidium bromide-resistant mutants were selected on DNA-free control plates.

**FIG 1:**
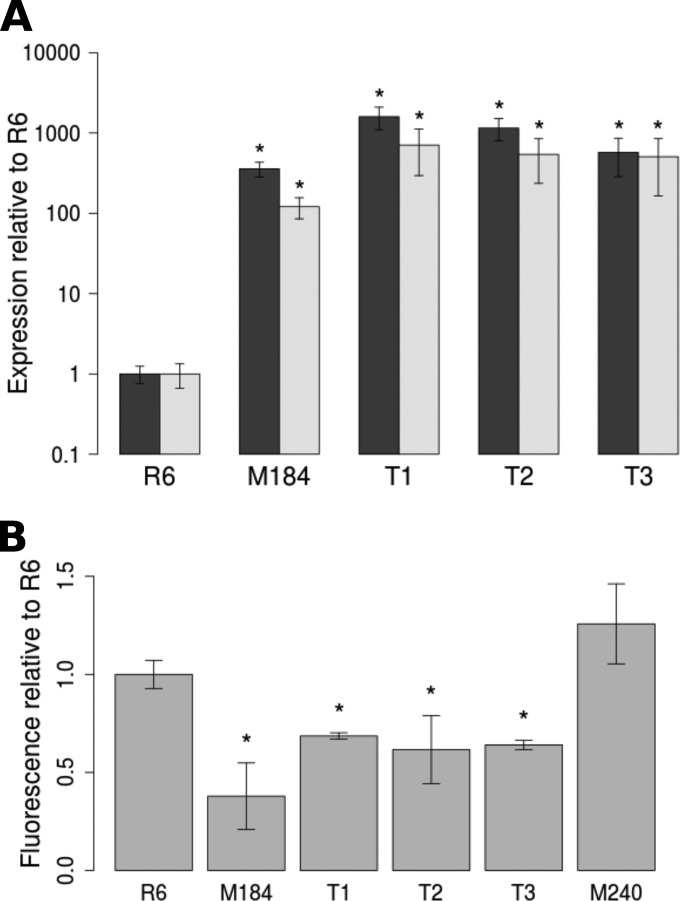
Expression of *patA* and *patB* and accumulation of ethidium bromide in M184 and R6^M184^ transformants. (A) Expression of *patA* (black bars) and *patB* (gray bars) relative to that of *rpoB*. (B) Accumulation of ethidium bromide after 10 min of incubation with 100 μM ethidium bromide. *, accumulation significantly lower than that of R6 (*P* < 0.05).

Three transformants, named R6^M184^ T1, R6^M184^ T2, and R6^M184^ T3, were chosen at random for further study. Expression of *patA* and *patB* in all three transformants was measured by quantitative real-time PCR to confirm successful transfer of the *patAB* overexpression phenotype. In M184 and all three transformants, both genes were expressed at a level 100 to 1,000 times higher than that in R6 (Fig. 1A).

The phenotypes of M184 and the three transformants were compared to confirm that the upregulation of *patAB* observed in the transformants conferred a phenotype similar to that of M184. First, the MICs of ciprofloxacin, norfloxacin, and ethidium bromide for R6, M184, the three transformants, and M240, an R6 derivative where *patB* has been inactivated by insertion of a *magellan2* minitransposon (7), were determined in the presence or absence of the ABC efflux pump inhibitor sodium orthovanadate. As expected, M184 and the three transformants showed decreased susceptibility to all three agents compared to that of R6 and M240, and this was reversible by addition of sodium orthovanadate (Table 3). However, the transformants differed slightly from M184, in that addition of sodium orthovanadate appeared to increase susceptibility to norfloxacin to a greater degree (at least 2 dilutions) in the transformants than in M184.

**TABLE 3:** MICs of ciprofloxacin, norfloxacin, and ethidium bromide for four R6^M184^ transformants in the presence and absence of sodium orthovanadate

Strain	MIC^*a*^ (mg/liter)					
	Cip	Cip + NaO	Nor	Nor + NaO	EtBr	EtBr + NaO
R6	0.5	0.25	1	<1	2	<1
M184	**2**	0.5	**8**	2	**16**	<1
R6^M184^ T1	**2**	0.25	**8**	<1	**16**	<1
R6^M184^ T2	**2**	0.25	**8**	<1	**16**	<1
R6^M184^ T3	**2**	0.25	**8**	<1	**16**	<1

a Cip, ciprofloxacin; Nor, norfloxacin; EtBr, ethidium bromide; NaO, 50 μM sodium orthovanadate. MICs are the modes from at least three independent experiments. Bold text indicates MICs that are 2 or more dilutions greater than the MIC for R6.

Second, the accumulation of ethidium bromide by R6, R6 *patB*::*magellan2*, M184, and the three transformants was also measured. After 10 min, all three transformants accumulated significantly lower levels of ethidium bromide than R6 (1.4- to 1.6-fold reduction compared to the level of accumulation for R6; *P* < 0.05; Fig. 1B). However, the decrease in ethidium bromide accumulation in the transformants was less pronounced than that in M184, which accumulated 2.6-fold lower levels of ethidium bromide than R6.

### Comparison of R6, M184, and transformants by whole-genome resequencing reveals a novel gene amplification.

To identify which mutation or combination of mutations was transferred from M184 to the three transformants to give *patAB* overexpression, the whole genomes of the transformants were sequenced by Illumina sequencing. Reads were aligned against the published R6 genome, and SNPs and small indels were identified as described above. Six mutations were found in transformant 1, three were found in transformant 2, and nine were found in transformant 3 (http://epapers.bham.ac.uk/1958/). Unexpectedly, however, there were no mutations shared between M184 and the three transformants, indicating that none of the 27 mutations found in M184 were the cause of *patAB* overexpression. This was confirmed by PCR and Sanger sequencing of all candidate mutations. It was then hypothesized that *patAB* overexpression was caused by a genomic rearrangement larger than that which is detectable using the SNP-calling approach. This was investigated in two ways.

**FIG 2:**
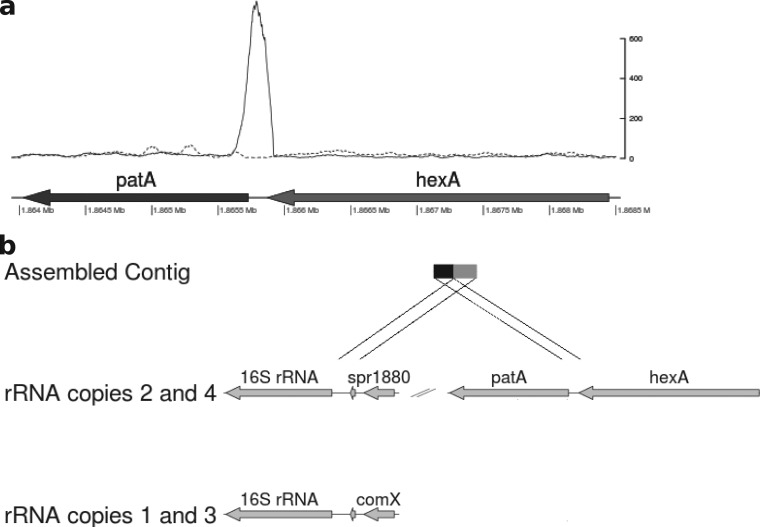
Evidence of genomic rearrangements from improperly paired reads. (a) Depth of reads aligned to the *hexA* and *patA* region following removal of properly paired reads by filtering for M184 (solid line) and R6 (dashed line). The *y* axis indicates read depth of improperly placed reads. (b) Matches found by a BLAST search between a contig assembled from improperly paired reads and the R6 genome.

First, the alignment of the reads against the R6 genome was examined for evidence of genomic rearrangements by identifying clusters of reads that did not align as proper pairs. Proper pairs are defined as reads aligning both within 250 bp of each other and in the correct relative orientation. Illumina sequence reads aligned against the published R6 genome were filtered to exclude all properly paired reads. Visual inspection of the filtered alignment identified a region with a high density of improperly paired reads upstream of *patA* in M184 and the transformants that was not present in R6 (Fig. 2a). The pairs of these reads were extracted from the raw data files by matching read names and realigned to the published R6 genome. They aligned to four regions of the genome, corresponding to an area just upstream of each of the four copies of the rRNA genes. A local assembly generated from these read pairs resulted in a single contig of 608 bp. This was used as a query sequence to search the published R6 genome sequence. Hits were found between the 3′ 326 bp of the contig and regions upstream of the rRNA loci, with the closest matches being with copies 2 and 4 (Fig. 2b). The remaining 282 bp matched to a region encompassing a region 183 to 464 bp upstream of *patA*. This suggested that a genomic rearrangement involving the *patA* region had occurred.

**FIG 3:**
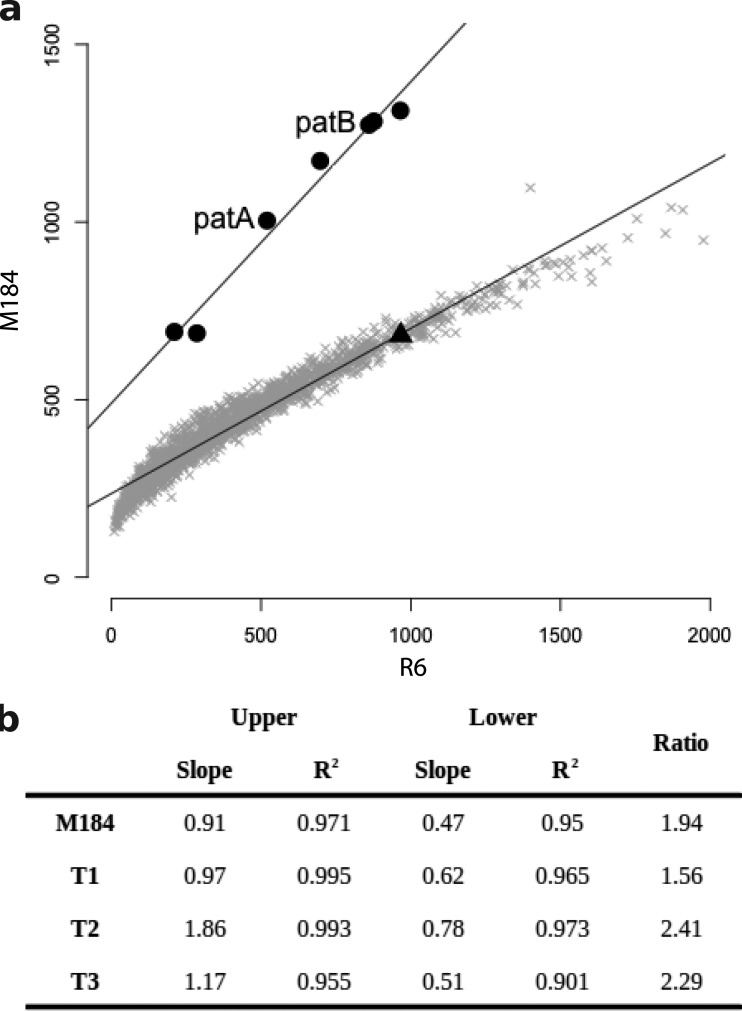
Evidence of duplication of a genomic region, including *patA* and *patB*, from comparison of per gene normalized read depths. (a) Per gene RPKM values for M184 plotted against those for R6. Six genes (filled circles) appeared to be present at a higher copy number than the remaining genes (gray crosses) in M184 than in R6. On further inspection, these genes were contiguous and included *patA* and *patB* (indicated). The *hexA* gene immediately upstream of *patA* (filled triangle) was not included in the higher-copy-number region. (b) The same six genes were present at increased copy numbers in M184 and the three R6^M184^ transformants (T1 to T3). The slope of the regression line calculated for the high-copy-number genes (upper) was divided by the slope of the regression line for the remainder of the genome (lower) to obtain an estimate of the copy number of the amplified genes.

Second, to look for evidence of changes in copy number of parts of the genome, normalized read depths, expressed as the number of reads per kilobase per million reads mapped (RPKM), were calculated for each annotated coding sequence (CDS) of more than 200 bp from the read alignments from each strain, and the profiles from M184 and the three transformants were compared against the profile from R6 (Fig. 3). This analysis identified six genes that had significantly higher read depths in M184 and the three transformants than in R6 (*P* < 0.05, one-tailed Student's *t* test). These genes were contiguous and corresponded to spr1880 to spr1887 (spr1883 was not included in the analysis, as it is less than 200 bp in length). The average RPKM ratio for these genes relative to that for R6 was 1.2 (standard deviation = 0.4) across all four *patAB*-overexpressing strains, whereas it was 0.6 (standard deviation = 0.1) for the rest of the tested CDSs. This region encompassed *patA* (spr1887) and *patB* (spr1885), and the final gene in the region, spr1880, is located directly upstream of the fourth rRNA locus. Taken together, these results suggest that a 9.2-kb region of the genome containing *patA* and *patB* has been tandemly duplicated in M184 and the three transformants.

To confirm the duplication suggested by the genome sequence analysis, PCR primers were designed to amplify the predicted novel junction between the two duplicated copies. PCR amplimers were obtained when DNA from M184 or the three transformants was used as a template but not when R6 DNA was used (http://epapers.bham.ac.uk/1958/). The sequences of the resulting PCR amplimers were determined and confirmed to be the same as the sequence of the contig assembled from the improperly paired reads.

To confirm that the two copies of the locus were arranged in tandem, a second PCR was carried out using primers specific for the 5′ end of *patA* and the 3′ end of the upstream gene *hexA*, which is not part of the duplication. In R6, these primers amplify the expected 357-bp region between *patA* and *hexA*. However, when DNA from M184 or the three transformants was used as a template and the PCR extension time was increased to 10 min, a second PCR amplimer of approximately 9 kb was observed (http://epapers.bham.ac.uk/1958/). This corresponds to amplification from the *patA* primer binding to the second copy of *patA*; it was absent when R6 DNA was used as a template.

### The gene amplification does not affect growth and has a low segregation rate.

The generation times of R6, M184, and the three transformants were measured during growth in liquid medium. M184 had a significantly longer generation time than R6 (46 ± 4 min compared to 34 ± 2 min; *P* < 0.05), but no significant growth defect was observed in the three transformants. This implies that carriage of the duplication itself does not affect the growth of R6 in liquid medium. We hypothesize that the growth of M184 is impaired because of one or more of the other mutations that it carries. For example, mutations were found in genes encoding proteins involved in key cellular processes, such as the translation factor EF-Tu (http://epapers.bham.ac.uk/1958/).

Large genomic duplications are often unstable, as recombination between the two copies of the duplicated locus can cause loss of the intervening DNA (2). The rate of loss of the duplication from M184 and the transformants was measured during growth in liquid medium in the absence of antibiotic. Following 12 h of exponential-phase growth in BHI broth without antibiotic, 72% ± 11% of the colonies tested had lost resistance to norfloxacin, suggesting a probability of loss per cell per generation of 0.018 ± 0.008 (*n* = 8). To confirm that the loss of norfloxacin resistance correlated with the loss of the duplication, seven norfloxacin-sensitive and seven norfloxacin-resistant colonies were tested by PCR for the presence of the duplication junction. PCR amplimers corresponding to the duplication junction were obtained from all seven of the resistant colonies but none of the sensitive colonies.

On the basis of these observations and under the assumption that continuous exponential growth occurs at a rate equivalent to that for R6 (generation time, 34 min), the proportion of cells carrying the duplication would drop below the detection limit of this experiment (5%) after 2.5 to 7 days.

### The duplication mechanism is unknown.

To determine the mechanism of gene amplification in M184, 50-bp sequences on either side of the start point and endpoint of the duplication and the duplication junction were aligned and examined for the presence of repeat sequences. When comparing the start point and endpoint of the duplication, no obvious repeat sequences were found. Only two identical bases (TA) were precisely conserved between the start and end of the duplication, and there were only 7 identical bases in total in the 20-bp region surrounding the join point (http://epapers.bham.ac.uk/1958/). Duplication of the region results in duplication of the conserved TA motif at the junction point, but introduction of gaps into the alignment to take this into account still did not reveal any larger conserved repeat sequences (data not shown).

Interestingly, the 9 bp of sequence on either side of the endpoint of the duplication formed a nearly perfect inverted repeat with only one mismatch (CTACAACAT-AAGTTGTAG). However, this sequence was not present in the sequence around the start of the duplication or at the duplication junction. The RegTransBase database of prokaryotic *cis*-regulatory elements (24) was searched for motifs similar to this inverted repeat using the STAMP motif alignment tool (25), but no matches were found.

Illegitimate end joining by DNA gyrase has been implicated in the formation of some gene amplifications, so the duplication start point and endpoint were examined for similarity to known pneumococcal DNA gyrase and topoisomerase IV cleavage site consensus sequences previously reported (26). No convincing matches were found.

### M184 also overexpresses *guaA* but not the other duplicated genes.

**FIG 4:**
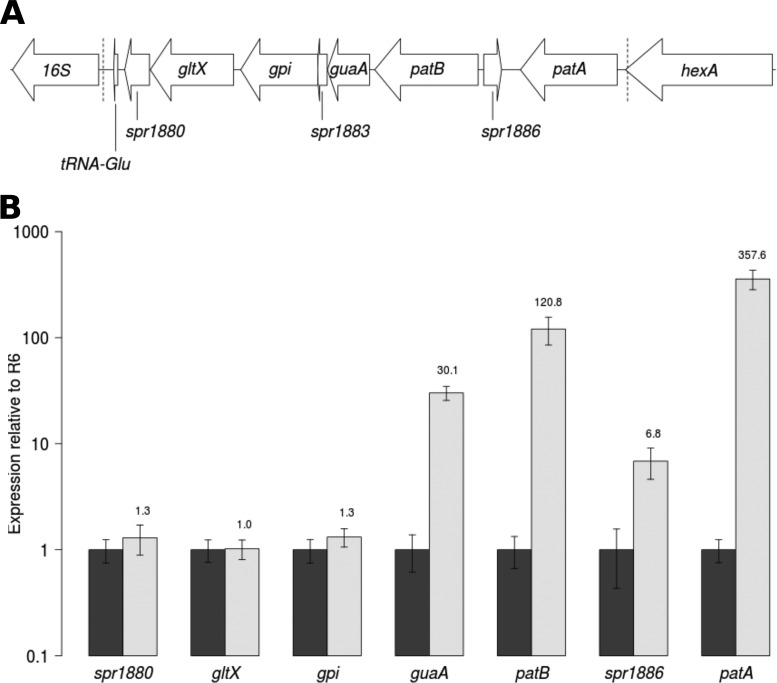
Measurement of expression of genes contained within the duplicated region of M184. (A) Representation of the genomic region around *patA* and *patB*. Dotted lines represent the extent of the duplicated region in M184 and the three transformants. (B) Expression of seven genes contained within the duplication measured by qRT-PCR from R6 (black bars) and M184 (white bars). Error bars represent the standard deviations from three biological replicates.

As well as *patA* and *patB*, the 9-kb duplicated region contains six other protein-coding genes and a tRNA gene, represented in Fig. 4A. The expression levels of five of these, spr1886, *guaA* (spr1884), *gpi* (spr1882), *gltX* (spr1881), and spr1880, from R6, M184, and the three transformants were measured by quantitative reverse transcription-PCR (qRT-PCR). In M184 and the three transformants, *guaA* was expressed at a level 20- to 50-fold higher than the level of expression in R6. This gene encodes a type 1 glutamine amidotransferase enzyme (NCBI conserved domain cd01745). Approximately 7-fold higher levels of transcript were observed in M184 for the degenerate transposase gene spr1886, which is located between the *patA* and *patB* genes in the opposite orientation. There was no difference in expression for any of the other tested genes (Fig. 4B). The expression levels of the tRNA gene could not be measured, as five copies of this gene exist in the R6 genome, meaning that unique primers could not be designed.

### Overexpression of *patA* and *patB* in M184 comes from the second copy of the locus.

Duplication of *patA* and *patB* did not fully explain the very high levels of *patAB* overexpression measured by qRT-PCR in M184 and the three transformants. Wild-type levels of expression from two identical gene copies could theoretically result in a 2-fold increase in expression, but instead, large (100- to 1,000-fold) increases in expression were observed.

There are two possible explanations for this observation. First, the duplication changes the genetic context of the second copy of *patA* and *patB*, which may result in increased expression of these genes due to the loss of regulatory control. Second, and alternatively, both copies of *patA* and *patB* could be overexpressed due to a positive-feedback loop if the initial increase of expression due to the duplication itself is sufficient to increase PatAB levels above a threshold value. If the first hypothesis is correct, inactivation of the first copy of *patA* should have no effect on the phenotype of M184, while inactivation of the second copy should reduce the level of efflux (and increase accumulation) to that for R6. On the other hand, if the second hypothesis is correct, inactivation of either copy of *patA* would reverse antibiotic resistance in M184.

To test this, M184 cells were transformed with donor DNA amplified from M246, a strain of R6 where *patA* is inactivated by insertion of the *magellan2* minitransposon (7). The donor PCR amplimer contained the *mariner* minitransposon flanked by an internal *patA* sequence, meaning that recombination with either copy of *patA* in M184 should be equally likely. It is known that multiple recombination events can occur simultaneously in one pneumococcal cell (27), so to select transformants where only one copy of *patA* was inactivated, cells were transformed with a series of dilutions of the PCR amplimer and candidates were selected from the lowest donor DNA concentration that produced viable colonies. It was expected that roughly equal numbers of transformants with the transposon inserted in each copy of *patA* would be obtained. However, a total of 52 spectinomycin-resistant transformants were obtained from three separate experiments, and all contained the *magellan2* transposon inserted in the first copy of *patA*. Three transformants containing the insertion in copy 1 of *patA* were selected at random for further testing. The MICs of ciprofloxacin, norfloxacin, and ethidium bromide were measured for these strains in the presence and absence of sodium orthovanadate. The MICs obtained for all three strains were identical to those for M184, suggesting that inactivation of the first copy of *patA* in M184 does not abolish PatAB overexpression.

### Overexpression from copy 2 of the *patAB* locus could be caused by read-through from a tRNA gene.

**FIG 5:**
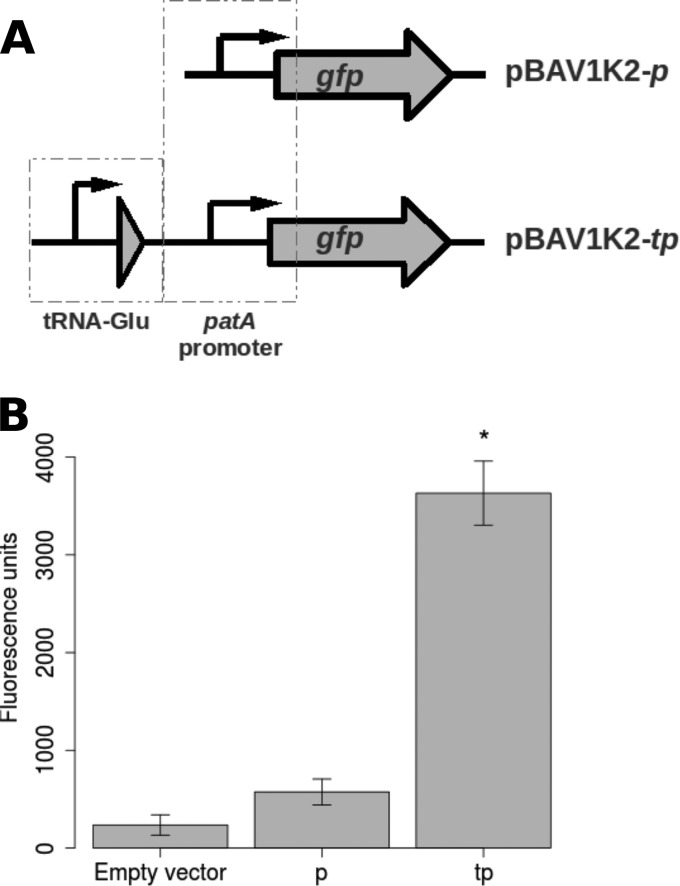
Measurement of activity of the *patA* promoter with and without the tRNA gene found upstream of *patA* copy 2 in strains containing the duplication. (A) Representation of the cloning of the tRNA gene and *patA* promoter upstream of a promoterless *gfp* gene in vector pBAV1K2. (B) Fluorescence levels measured from R6 cells containing pBAV1K2 (Empty vector), pBAV1K2*p* (p), and pBAV1K2*tp* (tp). Error bars represent the standard deviations from three biological replicates. *, fluorescence significantly greater than that from pBAV1K2*p* (*P* < 0.05, one-tailed Student's *t* test).

To determine why the second copy of *patA* appeared to contribute more to the M184 phenotype than the first copy, the genomic context of the second copy of *patA* was examined. The duplication results in the start codon of the second copy of *patA* being located 365 bp downstream of one of the five copies of a glutamate-specific tRNA gene found in the R6 genome. A list of S. pneumoniae transcriptional terminator predictions made by the TransTermHP program (28) was examined, and no Rho-independent terminator predictions were found in the downstream region of the tRNA gene. This suggested that increased expression of the second copy of the *patAB* locus could be conferred by transcriptional read-through from the tRNA gene. To test this prediction, a promoter probe plasmid was constructed by replacing a T5 promoter-*gfp* cassette from the broad-host-range plasmid pBAV1K-T5-*gfp* (22) with a promoterless gene coding for an unstable green fluorescent protein (GFP) variant from plasmid pMW82 (23), generating vector pBAV1K2. Unstable GFP was used to prevent changes in expression of GFP from the tested promoter from being obscured by fluorescence from accumulated stable GFP. Two different DNA fragments were cloned upstream of the promoterless *gfp* (Fig. 5A). The first contained the predicted *patA* promoter alone (pBAV1K2*p*), while the second, larger region spanned the M184 duplication junction from the end of copy 1 of spr1880 to the start of copy 2 of *patA* and contained the tRNA gene upstream of the *patA* promoter (pBAV1K2*tp*).

The promoter constructs were transformed into R6, and GFP fluorescence was measured during mid-logarithmic-phase growth (Fig. 5B). The fluorescence from pBAV1K2*p* was 2.4-fold higher than that from R6 harboring the empty pBAV1K2 vector (574 ± 132 and 236 ± 104 fluorescence units, respectively). The fluorescence levels observed for pBAV1K2*tp* were 6.3-fold higher than those observed for the *patA* promoter alone (3,682 ± 327 fluorescence units). However, this may be an underestimate of true promoter activity, as this strain had a severe growth defect compared to the growth of R6 containing pBAV1K2 or pBAV1K2*p* (generation times, 56 min and 35 min, respectively). Although it could not be confirmed, this may be a side effect of GFP toxicity.

## DISCUSSION

Overexpression of *patA* and *patB* confers clinically relevant resistance to fluoroquinolone antibiotics (9). In M184, overexpression of the *patA* and *patB* genes was conferred by a duplication of a 9.2-kb section of the genome and not a point mutation. To our knowledge, this is the first example of a large (>1-kb) gene amplification in S. pneumoniae. The only previously reported instance was an 18-bp duplication of a ribosome binding site in the *ermA* gene which caused macrolide resistance (29). Gene amplifications resulting in antibiotic resistance have been observed in several bacterial species and have been reviewed elsewhere (30).

Gene duplications can form by RecA-dependent and -independent mechanisms. RecA-dependent duplications are formed by homologous recombination between long repeats, where 25 to 40 nucleotides of sequence identity is required (31, 32). Common manifestations of this are recombinations between rRNA operons or insertion sequence elements (1, 33). Two mechanisms have been proposed to explain RecA-independent duplications. The first is strand slippage during rolling-circle DNA replication, which requires short areas of sequence identity (34). The second is illegitimate end joining during repair of double-stranded DNA breaks catalyzed by DNA gyrase (35). This does not require sequence homology at the duplication ends (36). No direct repeats were found at the ends of the duplicated region in M184, suggesting that the formation of this duplication is likely to be due to illegitimate end joining. Expression of *patAB* has previously been shown to be induced by fluoroquinolones and mitomycin C, which are DNA-damaging agents (8, 10). It is tempting to speculate that this could have been due to transient duplication promoted by DNA gyrase inhibition.

Similar homology-independent duplication junctions were previously observed in a study of 104 amplification mutants of Acinetobacter with an increased ability to grow on benzoate, where 36 of 104 distinct duplication junctions identified showed no sequence identity at the join point (37). Several of these homology-independent duplications were identified in multiple independent mutants, suggesting that homology-independent duplications can be formed in a site-specific manner. This could be explained by site specificity in DNA gyrase cleavage sites, as it has previously been shown that DNA gyrase cleaves preferentially at certain sites (38). In M184 the sites upstream and downstream of the duplication did not match the reported S. pneumoniae DNA gyrase consensus cleavage site (26). However, gyrase cleavage sites are highly degenerate, so the possibility of site-specific gyrase cleavage cannot be completely ruled out. It has also been suggested that site specificity in the formation and deletion of tandem repeats could be mediated by the DNA secondary or tertiary structure promoting recombination between particular sites through protein-protein or protein-DNA interactions (37, 39, 40). It is interesting that there is an inverted repeat motif at the 3′ end of the duplicated region that could be a binding site for an unknown protein factor.

Following an initial tandem duplication event, further gene amplification can occur by RecA-dependent homologous recombination between copies of the duplicated locus or by rolling-circle replication mechanisms (30). In several previous studies of adaptive gene amplification, observed copy numbers were greater than 2, indicating that further amplification has taken place. In Salmonella enterica, 5- to 40-fold amplification of genes encoding initiator tRNAs was shown to alleviate a fitness cost of resistance to peptide deformylase inhibitors (41). A 5-fold amplification of the *blaA* gene caused ampicillin resistance in Yersinia enterocolitica (42). In the Acinetobacter baumannii study described above, mutants with an increased ability to grow on benzoate were found to possess between 3 and 45 copies of the *cat* genes (37). Additionally, in a recent study, 10- to 110-fold amplifications of a Tn*6020* transposon containing an aminoglycoside resistance gene were shown to be the cause of tobramycin treatment failures in A. baumannii clinical isolates (43). A 4-fold amplification of the *folCEPBK* genes was found in Streptococcus agalactiae and resulted in decreased susceptibility to sulfonamides and trimethoprim (21). In these cases, the adaptive benefit of the amplification was either shown or assumed to be linked to the copy number of the amplified genes.

Further amplification of the duplicated region has not occurred in M184, and the copy number is therefore only 2. However, this duplication results in a level of expression of *patA* and *patB* much higher than that which can be explained by increased gene dosage alone. Insertional inactivation of the first copy of *patA* had no effect on the antibiotic resistance phenotype, indicating that the increased production of PatAB originates from the second copy of the locus. The duplication event alters the genomic context of the second copy of *patAB* and brings it under the control of the promoter of a tRNA gene.

Therefore, in contrast to previous studies, although the adaptive mutation in this strain is a gene amplification, the primary cause of the increased antibiotic resistance is altered expression of the *patAB* locus, rather than increased gene dosage. Antibiotic resistance mediated by promoter switching caused by a gene deletion event has been previously identified (44). However, to our knowledge, this is the first observation of a transient gene duplication causing antibiotic resistance due to placing resistance genes under the control of a highly active promoter. In the sulfonamide- and trimethoprim-resistant S. agalactiae isolate (21), expression of the second, third, and fourth copies of the *fol* genes was ascribed to an alternative promoter, but this led to only a 5-fold increase in gene expression, rather than the 4-fold increase that would be expected from gene dosage alone.

Most gene duplication events are unstable and are lost in the absence of selection due to recombination between identical copies of the locus (2). Typical segregation rates range from 0.01 to 0.15 loss events per cell per generation (2), although the 13.5-kb quadruplication event causing sulfonamide and trimethoprim resistance in S. agalactiae was highly stable and lost at a rate of only 0.003 per cell per generation (21). The duplication observed in M184 was unstable, as expected, and lost at an estimated rate of between 0.01 and 0.03 per cell per generation. Although this does not approach the level of stability observed in the S. agalactiae strain, this is still toward the more stable end of the range of previously observed segregation rates. The observed rate of duplication loss suggests that the duplication would be undetectable by the method used here within 2.5 to 7 days; however, this is assuming continuous exponential growth under laboratory conditions, whereas growth in a physiological situation may be limited by a number of factors. Therefore, it is possible that the stability of this duplication and, therefore, the increased expression of *patAB* could be higher in an environment in which antibiotics are used.

Overexpression of *guaA*, which is adjacent to *patB* in the S. pneumoniae genome, was also observed in M184 and the three transformants. This gene encodes a type 1 glutamine amidotransferase enzyme, which is one of a diverse family of enzymes catalyzing the transfer of ammonia from glutamine to a variety of substrates (45). However, overexpression of *guaA* was not observed in previous studies where expression of this gene was measured either as part of a global transcriptome analysis (10) or directly by qRT-PCR (46). The gene was overexpressed to a lesser extent than *patA* and *patB* (20- to 50-fold for *guaA* compared to 100- to 1,000-fold for *patA* and *patB*), so it is unclear whether this shows that *guaA* is coexpressed with *patAB* on a single transcript or whether the observed overexpression is due to leaky termination of transcription from *patB*. However, overexpression of *patAB* has been linked to DNA damage (8), and glutamine amidotransferases are known to be involved in nucleotide biosynthesis pathways (47); a functional link is therefore possible. The *gpi*, *gltX*, and spr1880 genes downstream of *guaA* were not upregulated in this study. Promoter prediction software (48) suggests the presence of a promoter sequence upstream of these genes (data not shown), so it is likely that they are expressed on at least one separate transcript.

The data presented here represent the first report of a large gene amplification in S. pneumoniae causing antibiotic resistance. This is also the first time that capture of a resistance gene by a strong promoter due to transient gene amplification has been observed. Taken together, these results add to the growing body of evidence suggesting that gene amplification is an important adaptive mechanism in bacteria allowing them to survive antibiotic exposure.
